# Discontinuation of hydroxychloroquine due to suspected retinal toxicity in systemic lupus erythematosus and the role of multimodal imaging

**DOI:** 10.55730/1300-0144.6035

**Published:** 2025-06-07

**Authors:** Burak İNCE, Mehmet Bedii OĞUREL, Zafer CEBECİ, Yavuz Burak TOR, Yasemin YALÇINKAYA, Ahmet GÜL, Murat İNANÇ, Bahar ARTIM-ESEN

**Affiliations:** 1 Division of Rheumatology, Department of Internal Medicine, Faculty of Medicine, İstanbul University, İstanbul, Turkiye; 2 Department of Ophthalmology, Faculty of Medicine, İstanbul University, İstanbul, Turkiye; 3 Department of Internal Medicine, Faculty of Medicine, İstanbul University, İstanbul, Turkiye

**Keywords:** Systemic lupus erythematosus, hydroxychloroquine, retinal disease, optical coherence tomography

## Abstract

**Background/aim:**

To evaluate the effect of hydroxychloroquine (HCQ) on disease activity and damage in patients with systemic lupus erythematosus (SLE) in whom HCQ had previously been discontinued due to retinal toxicity, and to examine and reevaluate toxicity findings through a detailed ophthalmological examination.

**Materials and methods:**

Patients with SLE who had been on HCQ for at least 3 years after achieving lupus low disease activity state (LLDAS) following remission induction, and were followed up for at least 3 years after HCQ discontinuation due to retinal toxicity diagnosed by visual field testing, were analysed. Disease activity, the number and severity of flares and damage were recorded whilst on HCQ and after cessation. All patients were examined by two ophthalmologists using multimodal imaging techniques to further analyse toxicity.

**Results:**

Sixty-one patients (age at diagnosis 33.4 ± 10.5, 88.5% female) were included. The percentage of visits maintaining LLDAS was significantly higher during HCQ treatment (p = 0.001). A significant number of patients experienced flares after HCQ discontinuation, with the mild–moderate type predominating (p = 0.006 and p = 0.026). Mean damage scores were higher at the end of the study period (p = 0.001). In the ophthalmologic examination (mean duration after drug cessation: 70.3 ± 52.3 months), signs of HCQ toxicity were not detected in 40 patients (65.6%), and HCQ was reinitiated for these patients. Of 21 (34.4%) patients who had visual field defects in reexamination, only five (8.2%) had typical retinal toxicity by multimodal imaging. Sixteen (26.2%) patients had macular atrophy due to other causes.

**Conclusion:**

Hydroxychloroquine is effective in controlling disease activity and preventing damage in SLE, and the opportunity for remedication is valuable. More than half of the patients who could restart HCQ after reexamination show the importance of performing multimodal imaging to diagnose retinal toxicity and to distinguish macular pathologies with different aetiological background.

## 1. Introduction

Hydroxychloroquine (HCQ) is an immunomodulatory drug shown to be effective in a variety of autoimmune diseases. In systemic lupus erythematosus (SLE), it is effective in patients with arthritis and cutaneous involvement. Furthermore, it has been shown to control overall disease activity, allow steroid tapering, reduce organ damage, and improve survival in multiple studies [1–5]. Its additional antithrombotic, antihyperlipidaemic, and glucose-lowering effects make it a highly beneficial drug in this group of patients. In previous studies, it was shown that blood levels of HCQ were negatively correlated with disease activity, and withdrawal of treatment was associated with disease flares [6, 7]. Adverse effects also play an important role in the cessation of treatment.

A prominent long-term complication of HCQ is retinal toxicity. Drug accumulation within the retinal pigment epithelium, inhibition of lysosomal functions, and retention of lipofuscin—which is associated with photoreceptor degeneration—and inhibition of the reisomerisation of all-trans-retinol, which is important in the visual cycle, are among the proposed mechanisms of HCQ retinal toxicity [8, 9].

In studies published before 2010, the estimated risk of HCQ retinopathy was found to be < 2% using conventional methods and visual field tests [10, 11]. The implementation of modern screening and multimodal imaging techniques in the last decade, such as optical coherence tomography (OCT) and fundus autofluorescence, has increased the recognition of retinal pathologies and the frequency of retinopathy reported in cohort studies [12, 13]. The overall prevalence of retinopathy has been reported to be as high as 7.5% in different studies after 5 years of use, with variations depending on daily dosage [14–16]. It has been reported that changes observed in the early stages of toxicity are usually asymptomatic and may not be detectable by conventional examination [14]. On the other hand, considering the limited treatment options for SLE and the substantial role of HCQ, discontinuation should be based on solid evidence from the physician’s side, and the assessment of toxicity should be undertaken cautiously. While these screenings were previously performed according to the capabilities of the centres, current guidelines recommend annual examinations with visual field tests and OCT in patients who have been using HCQ for more than 5 years. Periodic reexamination after cessation of therapy is also recommended in patients with retinopathy, until retinal findings stabilise [17].

In this study, we sought to determine the effect of HCQ on disease activity and damage in patients with SLE in whom HCQ had previously been discontinued due to retinal toxicity. Furthermore, due to the absence of a gold-standard method, we aimed to assess the toxicity findings using multimodal imaging techniques and to evaluate the performance of these methods.

## 2. Patients and method

The records of patients followed in the SLE/antiphospholipid syndrome clinic of a tertiary care centre between January 1995 and December 2020 were reviewed. Patients older than 18 years who fulfilled the Systemic Lupus International Collaborating Clinics (SLICC) classification criteria, had been on hydroxychloroquine (HCQ) for at least 3 years after achieving lupus low disease activity state (LLDAS), and had been followed for at least 3 years after HCQ discontinuation due to retinal toxicity diagnosed by visual field testing were included in the study. Patients with ocular involvement of SLE (optic neuritis, retinal vasculitis) or total vision loss in any eye were excluded. The standard protocol used for the patients registered after 1991 in the weekly SLE clinic consisted of data on demographic characteristics, SLE classification criteria (American College of Rheumatology, [ACR] and Systemic Lupus International Collaborating Clinics [SLICC]), autoantibody profile, treatment history, antiphospholipid syndrome (APS; classification criteria), features of nephritis including histopathology when available, SLICC/ACR damage index, treatment history, and mortality. For this study, demographic characteristics, cumulative clinical features, and data on SLICC classification criteria were retrieved from the database and revised. The estimated glomerular filtration rate (eGFR) was calculated using the “Chronic Kidney Disease Epidemiology Collaboration (CKDEPI)” equation, which was based on patient data [18]. Disease activity and both the number and severity of flares were recorded for each patient during the 3 years preceding HCQ termination and the first 3 years after cessation of the drug (Figure). Damage scores were calculated both at the time HCQ was stopped and at the end of the third year following drug withdrawal. SLE Disease Activity Index 2000 (SLEDAI-2K) and LLDAS were implemented retrospectively for every 3 months to evaluate disease activity [19, 20]. Flares were defined according to SLEDAI-2K.

All patients were examined by two experienced ophthalmologists (ZC, MBO) and assessed using computerised visual field testing, optical coherence tomography (OCT), and fundus autofluorescence (FAF) to further analyse retinal toxicity. The visual field was assessed by automated static visual field testing using a Humphrey computerised visual field device (Carl Zeiss Meditec Inc., Dublin, CA, USA), after applying refractive correction appropriate for the test distance determined prior to pupil dilation. Optical coherence tomography was conducted using a Spectral-domain OCT (SD-OCT) (Spectralis; Heidelberg Engineering GmbH, Heidelberg, Germany) device to acquire a horizontal scan with 25-line raster passing through the macula. Central macular thickness was measured and the foveal, parafoveal, and perifoveal regions were evaluated for losses in the outer retinal layers, losses in the photoreceptor inner segment/outer segment (IS/OS) band, and RPE irregularity/loss. Fundus autofluorescence images were also obtained with the Spectral-domain OCT (SD-OCT) (Spectralis, Heidelberg Engineering GmbH, Heidelberg, Germany) device in a 55° field of view, and were evaluated for anomalies in terms of hypo- and/or hyperautofluorescent areas in the parafoveal and perifoveal regions. Retinal toxicity was defined primarily based on OCT findings, including disruption of the ellipsoid zone, external limiting membrane defects, and decreased retinal thickness; FAF findings were regarded as supportive but not mandatory. Macular changes or atrophy on fundus examination were also considered.

The study was conducted in accordance with the Declaration of Helsinki and was approved by the Istanbul University, Istanbul Faculty of Medicine Ethics Committee (Date-number: 2021-89942). IBM SPSS Statistics for Windows, version 22.0 (IBM Corp., Armonk, NY, USA), was used for statistical analyses. Continuous variables are presented as the mean (SD) or median (IQR), whereas categorical variables are presented as number and percentage. For categorical comparisons, the chi-square test and Fisher’s exact test were used. Student’s t-test and Mann–Whitney U test were used to compare continuous variables, flare counts, and damage data, according to the normality of the data. The McNemar test was used to compare the proportions of patients with flares. A p-value less than 0.05 was accepted as statistically significant.

## 3. Results

Out of 88 (9.3%) patients with recorded HCQ retinal toxicity in a cohort of 950 patients with SLE, 61 patients with complete follow-up data and ophthalmological reexamination results were included in the analyses. The mean age of the patients at SLE diagnosis was 33.4 ± 10.5 years (range: 10–57), and 88.5% were female. Five patients (8.2%) also had antiphospholipid syndrome. The median total follow-up time was 174 (97) months. Cumulative clinical features of patients, according to the SLICC classification criteria, are as follows: cutaneous lupus in 34 (55.7%), mucosal ulcers in six (9.8%), alopecia in 20 (32.8%), arthritis in 43 (70.5%), serositis in 11 (18%), renal involvement in 25 (41%), neurological involvement in three (4.95%), haemolytic anaemia in five (8.2%), leukopenia in 13 (21.3%), thrombocytopenia in nine (14.8%), anti-dsDNA positivity in 36 (59%), and low complement levels in 22 (36.1%).

The average duration of HCQ treatment was 96 (71) months, and the mean follow-up time was 48 (52) months after the drug was discontinued. The maximum HCQ dose was 5 mg/kg per day. Hypertension was present in 24 (39.3%) patients, and diabetes mellitus was present in nine (14.8%) patients.

### 3.1. Disease activity

Comparison of mean disease activity in the 3-year period when patients were on HCQ with the 3 years postcessation revealed a significantly lower mean SLEDAI-2K score in the former (0.89 ± 1.28 versus1.3 ± 1.6, p = 0.02). The percentage of visits maintaining LLDAS was significantly higher during the 3 years on HCQ treatment compared to the 3 years postcessation (89.7 ± 17.6 versus 80.1 ± 23.5, p = 0.001).

### 3.2. Flares

We detected a total of 64 flares in 35 patients over a 6-year period. A significantly higher number of patients experienced flares after HCQ was stopped compared to the period when patients were on HCQ treatment (30 [49.2%] versus 18 [29.5%], p = 0.017). The mean number of total flares was also higher after cessation of HCQ (0.72 ± 0.9 versus 0.31 ± 0.5, p = 0.001). Examining the breakdown of flares after cessation of therapy according to severity, we found that the mean number of mild–moderate flares was significantly higher after cessation (0.57 ± 0.8 versus 0.26 ± 0.48, p = 0.035), whilst serious flares were also more common in the postdiscontinuation period but without statistical significance (0.16 ± 0.42 versus 0.05 ± 0.22, p = 0.067). Active skin disease was more frequently observed during flares after drug cessation (mean 0.2 ± 0.4 versus 0.06 ± 0.25, p = 0.006). Table 1 shows detailed data regarding the number of patients who experienced flares.

### 3.3. Damage

A total of 39 patients had damage in at least one domain except retinal changes. A significantly higher number of patients had damage in at least one damage item (excluding retinal changes) at the end of the 3 years after drug cessation compared to the visit when HCQ was stopped (29.5% versus 64.9%, p < 0.001). Comparison of the mean damage score, after exclusion of retinal changes, at the beginning and the end of the 3-year period without HCQ revealed a significantly higher score in the latter (0.4 ± 0.7 versus 1.08 ± 1.05, p < 0.001). All damage scores are provided in Table 2. A higher frequency of myocardial infarction was observed at the end of the 3 years following HCQ cessation.

### 3.4. Ophthalmologic examination

Ophthalmologic reexamination was performed 70.3 ± 52.3 months after drug cessation, and no signs of toxicity were observed in forty (65.6%) patients. Although 21 (34.4%) patients had visual field defects in the last examination, multimodal imaging with OCT and FAF revealed that only five (8.2%) patients had typical retinal toxicity. Sixteen (26.2%) patients (32 eyes) were found to have macular atrophy due to causes other than HCQ toxicity. Macular pathology was related to macular degeneration in six patients, central serous chorioretinopathy (CSCR) in eight patients, epiretinal membrane in one patient (one eye), vitromacular traction in one patient (one eye), and high myopia in one patient. Since the discrimination of macular pathology and follow-up would not be possible with multimodal imaging in these patients, HCQ was not reinitiated.

Data regarding multimodal imaging in patients who were previously diagnosed with retinal toxicity but could restart HCQ compared to those who could not, are shown in Table 3. Macular atrophy on fundus examination, hypofluorescence with surrounding rim of hyperfluorescence on FAF, and ellipsoid zone, external limiting membrane defect, and decreased retinal thickness in OCT were the main findings associated with retinal toxicity. Receiver operating characteristic (ROC) curve analysis showed that retinal thickness was a significant discriminator of toxicity, with an area under curve (AUC) of 0.802 (p = 0.002), and a threshold of ≤ 231 μm provided 80% sensitivity and 93.7% specificity in eyes with retinal toxicity.

Comparison of patients with and without retinal toxicity showed that the frequency of hypertension was significantly higher in patients with retinal toxicity (100% versus 95%, p = 0.001) (Table 3). Age, frequency of diabetes, duration of HCQ treatment, and duration after cessation of HCQ treatment were not different between groups.

## 4. Discussion

We showed the effect of HCQ in lowering disease activity, preventing relapses and damage in our patients with SLE, and demonstrated that reinitiation of HCQ was possible after multiple examination methods in patients who were previously diagnosed with retinal toxicity.

Lupus low disease activity state (LLDAS) is a recently developed treat-to-target tool for patients with SLE, and among its definitions are low disease activity according to SLEDAI and low-dose corticosteroids (CS) [20]. Hydroxychloroquine’s positive effect at reaching LLDAS was shown previously in a study consisting of 30 patients with SLE, similar to our study [21]. In this study, 80% of patients who received HCQ for more than 104 weeks reached LLDAS, compared to 10% at baseline. Disease activity according to SLEDAI, the maintenance dose of CS, and serological parameters such as anti-dsDNA titres were shown to be improved. Similarly, we found that patients off HCQ treatment had higher SLEDAI scores and were less likely to maintain LLDAS, which supports the importance of the drug in SLE treatment.

Our study also shows that after cessation of HCQ, the frequency of mild-to-moderate flares and cutaneous symptoms was higher, in parallel with previous studies. Although the number of severe flares was also higher in this period, the difference did not reach statistical significance probably due to the low number of patients. In a recent SLICC study, the hazard rate of relapse in patients who discontinued HCQ treatment was 1.56, compared to patients on HCQ maintenance. During disease flares, the need for an increase in immunosuppressants was more frequent compared to the maintenance group (72% versus 53.1%) but hospitalisation due to flares was uncommon [22]. On the other hand, a historical study conducted by the Canadian Hydroxychloroquine study group showed that withdrawal of HCQ caused an increased risk of severe flares, up to 6.1 times higher compared to the treatment group [6].

Another important finding of our study is the significant increase in damage after cessation of HCQ in patients. In particular, there was a striking difference in the frequency of myocardial infarction during this period. HCQ’s protective effect on damage accrual was also shown in multiple cohort studies. In a large cohort study consisting of 2054 patients, Petri et al. showed that past use of hydroxychloroquine had a preventive effect on damage accrual [23]. Furthermore, in a systemic literature review that included data of 481 patients with SLE, HCQ was found to be the only variable significantly associated with less damage accrual [24]. The possible effect of HCQ in preventing cardiovascular events (CVE) is a remarkable finding. A case-control study involving 10,268 patients with SLE and rheumatoid arthritis similarly showed that HCQ use was associated with a reduced risk of CVE [25]. However, continuous CS treatment and older age are well-known risk factors for CVE in patients with SLE, and caution is needed when interpreting the damage data at the end of the study period [26].

The low prevalence of true retinal toxicity (0.5%) in our cohort is consistent with a recent study that reported the probability of retinal toxicity to be less than 1% until 10 years of HCQ use at doses < 5 mg/kg [27]. Hypertension was the only variable significantly associated with retinopathy, although it was not reported as a potential risk factor in larger cohort studies [28]. Patients with true toxicity had a numerically longer median duration of HCQ treatment in our study; however, this difference did not reach statistical significance, possibly due to the small number of patients. Over 10 years of HCQ exposure should alert physicians to the need for regular retinal examinations to check for retinal toxicity. In our study, the average drug-free duration was identical for patients with persistent retinal toxicity and those with no pathological retinal changes at the final examination. Similarly, a previous study showed that sustained visual improvement was not observed after cessation of HCQ [29]. As our final examination results enabled us to consider restarting the treatment in some patients, it may be possible that some of them had early findings of retinal toxicity that regressed after cessation, or the decision to stop the drug was based only on visual field testing and fundus examination, which led to misdiagnosis. Previous publications on regression of HCQ retinopathy are limited to case reports and series [30–32]. Mititelu et al reported outer retinal regeneration in five patients with HCQ toxicity two of whom also had visual improvement after drug cessation [31]. In another study, out of 12 patients with long-term HCQ use evaluated by longitudinal multifocal electroretinography (ERG), two were found to have improved results after treatment termination [32].

The decision to discontinue HCQ treatment should not be based solely on fundus examination and bilateral visual field testing. Ophthalmic multimodal imaging is very informative for the detection of HCQ toxicity and should be used to decide to stop the drug. In our study, perifoveal loss of the ellipsoid zone and external limiting membrane defects on OCT were considered decisive for retinal toxicity, and OCT findings did not correlate with fundus examination. Significantly reduced macular thickness on OCT in eyes with true toxicity was consistent with these findings. ROC analysis further supported the association between reduced macular thickness and toxicity, suggesting that it may help in the assessment of HCQ retinopathy. Drusen are extracellular deposits composed of lipids, proteins and cellular debris that accumulate between the retinal pigment epithelium (RPE) and Bruch’s membrane. They are typically seen as yellowish lesions on fundoscopic examination and are most commonly associated with aging and age-related macular degeneration (AMD) [33]. The presence of drusen is not considered a typical or direct manifestation of HCQ retinopathy, and similarly, it was not observed in our study among patients diagnosed with retinal toxicity.

Fundus autofluorescence demonstrated areas of hypofluorescence bordered by a rim of increased autofluorescence, indicating ongoing damage, in all patients with true toxicity. There was only one patient with abnormal FAF results but normal OCT, and this patient was not considered to have retinal toxicity. Although sensitivity and specificity analyses could not be reliably performed due to the limited number of patients, we believe that OCT has a higher specificity than fundus examination and FAF in detecting retinal toxicity based on these observations. This interpretation aligns with previous reports suggesting that FAF serves as a supportive, but not primary diagnostic method for retinal toxicity [12]. Furthermore, our findings suggest that conventional fundus examination was the least reliable method in detecting early or subtle signs of toxicity.

Various conditions can contribute to the development of macular pathology, making eye examination advisable before initiating HCQ treatment. In our cohort, age-related macular degeneration and CSCR were prominent among macular pathologies other than HCQ retinopathy. Therefore, initial ophthalmologic evaluation is particularly important for individuals at higher risk, especially those of advanced age or with a history of high-dose steroid use.

Considering the benefits of treatment and the low potential for serious side effects, it is reasonable to think that every patient without absolute contraindications should use HCQ. Treatment was restarted only in patients with no signs or findings of retinal toxicity on ophthalmologic investigations with multimodal imaging techniques.

Our study has some limitations. Data regarding clinical features and initial ophthalmologic examination were collected retrospectively. Some details were missing, and the decision regarding HCQ retinal toxicity was based only on visual field testing. However, the limited availability of technical resources at the time was different from the current situation. Regarding the effect of HCQ on disease activity, the statistical method used was not ideal; however, confounders were considered where possible. Additionally, considering that severe clinical manifestations such as lupus nephritis are more commonly observed in the early years of the disease, the mean follow-up period of over 10 years in our study may have contributed to a relatively lower frequency of severe flares. However, the main objective of this study was to find out whether termination of HCQ was based on rational grounds and whether reinitiation was possible after appropriate examination methods. On the other hand, despite the limitations, patient assessment and follow-up were performed by the same experienced rheumatologists over a long period at a dedicated lupus outpatient clinic. Moreover, the final ophthalmologic examination was conducted by the same experienced ophthalmologists using multiple imaging techniques.

In conclusion, our findings support the well-known beneficial effects of HCQ in patients with SLE. Therefore, the decision to discontinue HCQ treatment should be based on the results of multimodal imaging techniques. Since macular pathology can have a different aetiological background, an initial ophthalmologic examination before initiating HCQ is also necessary. Whether HCQ retinal toxicity is reversible at an early stage is unknown, and reevaluation after cessation of the drug may provide an opportunity to reinitiate treatment.

## Figures and Tables

**Figure:**
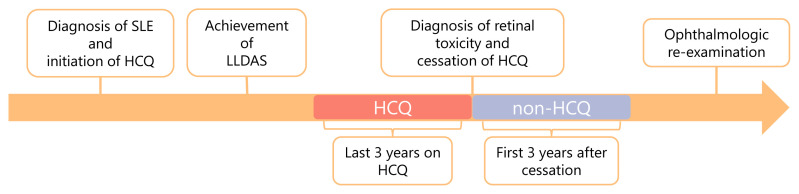
Timeline illustrating the study methodology.

**Table 1:** Evaluation and comparison of flares in 61 patients with SLE before and after HCQ discontinuation. The McNemar test was used to compare the proportions of patients with flares.

	No. of patients experienced flare				p
	Under treatment		After cessation		
	n	%	n	%	
**Total flares**	**18**	**29.5**	**30**	**49.2**	**0.017**
**Mild-moderate flares**	15	24.5	24	39.3	0.06
**Severe flares**	3	4.9	9	14.8	0.14
**Cutaneous findings**	**4**	**6.6**	**13**	**21.3**	**0.012**
**Pleuritis**	5	8.2	2	3.3	0.45
**Arthritis**	9	14.8	14	23	0.32
**Fever**	2	3.3	4	6.6	0.68
**Neurologic**	0	0	1	1.6	0.62
**Vasculitis**	1	1.6	3	4.9	0.62
**Nephritis**	1	1.6	3	4.9	0.3
**Myositis**	0	0	0	0	1
**Autoimmune hemolytic anemia**	3	4.9	2	3.3	0.57
**Thrombocytopenia**	1	1.6	1	1.6	1
**New immunosuppresive treatment and/or hospital admission**	10	16.4	14	23	0.5
**Increase in prednisone <0.5 mg/kg**	13	21.3	21	34.4	0.13
**Increase in prednisone >0.5 mg/kg**	4	6.6	7	11.5	0.5

**Table 2:** Evaluation and comparison of damage findings according to the SLICC/ACR damage index. AVN: avascular necrosis; CABG: coronary artery bypass graft; CVE: cerebrovascular event; PNP: peripheral neuropathy; GFR: glomerular filtration rate; MI: myocardial infarction. Chi-square and Fisher’s exact tests were used.

	At the time of HCQ cessation		3 years later after HCQ cessation		p	OR (95% CI)
	n	%	n	%		
**Patients with at least one damage item**	**18**	**29.5**	**39**	**63.9**	**<0.001**	**2.1 (1.4–3.3)**
**Ocular**						
**Cataract**	1	1.6	3	4.9	0.3	
**Neuropsychiatric**						
**CVE**	0	0	1	1.6	0.3	
**PNP**	0	0	1	1.6	0.3	
**Renal**						
**GFR<50 mL/min**	2	3.3	4	6.6	0.4	
**Proteinuria (>3.5 g/24 h)**	0	0	1	1.6	0.3	
**Pulmonary**						
**Pulmonary fibrosis**	2	3.3	3	4.9	0.65	
**Cardiovascular**						
**CABG**	1	1.6	1	1.6	1	
**MI history**	**0**	**0**	**4**	**6.6**	**0.04**	**0.9 (0.87–0.99)**
**Valvular disease**	3	4.9	6	9.8	0.3	
**Pericarditis**	0	0	1	1.6	0.3	
**Peripheral vascular**						
**Tissue loss**	0	0	3	4.9	0.08	
**Musculoskeletal**						
**Osteoporosis**	4	6.6	9	14.8	0.14	
**AVN**	4	6.6	7	11.5	0.34	
**Osteomyelitis**	0	0	1	1.6	0.3	
**Deforming arthritis**	2	3.3	4	6.6	0.4	
**Others**						
**Diabetes**	5	8.2	11	18	0.1	
**Malignancy**	1	1.6	1	1.6	1	

**Table 3:** Clinical, examination and imaging findings in patients with true retinal toxicity vs. those who restarted the drug after cessation. (Patients with other macular pathologies who did not restart treatment were excluded). Fundus examination and imaging were performed in 90 eyes. BVF: binocular visual field; ELM: external limiting membrane; OCT: optical coherence tomography; RPE: retinal pigment epithelium. Chi-square and Fisher’s exact tests were used.

	Patients with no signs of retinal toxicity (n = 40) n (%)	Patients with true retinal toxicity (n = 5) n (%)	p	OR	95% CI
Sex (F)	38 (95)	5 (100)	0.61		
Age (mean)	47 ± 12.6	54.5 ± 10.5	0.21		
Duration of HCQ treatment (median)	96 (89)	164 (190)	0.16		
Duration after cessation of HCQ (median)	50 (59)	48 (88)	0.54		
**Hypertension**	**11 (27.5)**	**5 (100)**	**0.001**	**1.45**	**1.05–2**
Diabetes	5 (12.5)	2 (40)	0.16		
Chronic renal insufficiency (eGFR< 50 mL/min)	3 (7.5)	1 (20)	0.35		
**Fundus examination (performed in 90 eyes)**					
**RPE changes**	**18(22.5)**	**6 (60)**	**0.01**	**5.16**	**1.3–20.3**
Drusen	4 (5.1)	0(0)	0.3		
RPE atrophy + drusen	**0(0)**	**2 (20)**	**0.01**	**1.25**	**1–1.7**
Macular atrophy	**0(0)**	**2 (20)**	**0.01**	**1.25**	**1–1.7**
**BVF defects**	**34(42.5)**	**10(100)**	**0.001**	**5.75**	**1.02–37.3**
**Fundus autoflorescence findings (performed in 90 eyes)**					
Hypoflorescence	1 (1.3)	0(0)	0.7		
Hypo and hyperfluorescence	2(2.5)	10(100)	**<0.001**	**351**	**28.9–4270**
**OCT findings (performed in 90 eyes)**					
Elipsoid zone and ELM defect	**0(0)**	**10(100)**	**<0.001**	**308**	**25–3784**
Macular thickness	**259 ± 24.1**	**228.1 ± 36.3**	**0.002**		

## Data Availability

Datasets of the study are available from the corresponding author on reasonable request.
